# Sphingosine-1-Phosphate Receptor 1, Expressed in Myeloid Cells, Slows Diet-Induced Atherosclerosis and Protects against Macrophage Apoptosis in *Ldlr* KO Mice

**DOI:** 10.3390/ijms18122721

**Published:** 2017-12-15

**Authors:** Leticia Gonzalez, Alexander S. Qian, Usama Tahir, Pei Yu, Bernardo L. Trigatti

**Affiliations:** Department of Biochemistry and Biomedical Sciences, and Thrombosis and Atherosclerosis Research Institute, McMaster University, Hamilton, ON L8L 2X2, Canada; leticia.gonzalezjara@gmail.com (L.G.); qiana@mcmaster.ca (A.S.Q.); usama_mayo@hotmail.ca (U.T.); yupei0317@gmail.com (P.Y.)

**Keywords:** apoptosis, atherosclerosis, HDL, macrophage, mouse model, sphingosine-1-phosphate receptor 1

## Abstract

We generated myeloid specific sphingosine-1-phosphate receptor 1 (*S1pr1*) deficient mice by crossing mice that had myeloid specific expression of Cre recombinase (*lyzM^Cre^*) with mice having the *S1pr1* gene flanked by loxP recombination sites. We transplanted bone marrow from these mice and control *lyzM^Cre^* mice with intact macrophage *S1pr1* gene expression into low-density lipoprotein (LDL) receptor gene (*Ldlr*) deficient mice. The resulting chimeras were fed a high fat atherogenic diet for nine or twelve weeks and evaluated for atherosclerosis development in the aortic sinus. Selective *S1pr1* deficiency in bone marrow-derived myeloid cells resulted in accelerated development of atherosclerosis, necrotic core formation and the appearance of apoptotic cells within atherosclerotic plaques of *Ldlr* knockout mice in response to a high fat diet. Examination of macrophages in culture revealed that the sphingosine-1-phosphate receptor 1 selective agonist, SEW2871 or high density lipoprotein (HDL), protected macrophages against apoptosis induced by endoplasmic reticulum (ER) stress or oxidized LDL, through activation of phosphatidylinositol-3-kinase/Akt signaling. Targeted *S1pr1*-deletion prevented Akt activation and protection against apoptosis by either SEW2871 or HDL. Our data suggests that sphingosine-1-phosphate receptor 1 in macrophages plays an important role in protecting them against apoptosis in vitro and in atherosclerotic plaques in vivo, and delays diet induced atherosclerosis development in *Ldlr* deficient mice.

## 1. Introduction

Atherosclerosis is a chronic inflammatory disease characterized by the accumulation of lipids and inflammatory cells in the walls of large- and medium-sized arteries [1]. The accumulation of apoptotic macrophages from both increased apoptosis and defective clearance of apoptotic debris contributes to the growth of necrotic cores within atherosclerotic plaques [2,3,4,5,6,7]. This plays an important role in the transition from stable to unstable plaques, which are more prone to rupture and are associated with myocardial infarction and stroke [2,3,4,5,6,7]. Oxidized low density lipoprotein (oxLDL) exhibits diverse pro-atherogenic properties, promoting monocyte recruitment, limiting macrophage egress from the arterial wall and promoting macrophage cell-death, which can lead to necrotic core formation and appears to be a key mediator of atherosclerosis initiation and progression [8,9,10,11]. Furthermore, the accumulation of unesterified cholesterol in the endoplasmic reticulum (ER) of macrophage foam cells disrupts normal ER function and induces ER-stress, markers of which have been observed at all stages of atherosclerotic plaque development in both humans and in mouse models [12,13,14,15,16]. ER stress leads to activation of apoptosis in macrophages through mechanisms involving prolonged activation of the unfolded protein response, expression of the transcription factor, CHOP/GADD153 and activity of the pro-apoptotic factor Bim [17,18].

The sphingosine 1 phosphate receptor 1 (S1PR1) is one of 5 G-protein coupled receptors (S1PR 1–5) that signal in response to sphingosine-1-phosphate (S1P). S1PR 1–5 exhibit different patterns of expression in different cell types [19]. S1P has been reported to protect macrophages from apoptosis, to modulate macrophage trafficking and also to favor an anti-inflammatory macrophage phenotype [20,21]. Interference with S1P signaling in vivo has been reported to affect atherosclerosis development in mouse atherosclerosis models. For example, reduction of S1P levels through inhibition of sphingosine kinase 1 increases atherosclerosis development, while elevation of S1P levels through selective inactivation of S1P lyase in hematopoietic cells protects against atherosclerosis [22,23]. Treatment of atherosclerosis-prone LDL receptor gene knockout (*Ldlr* KO) or apolipoprotein E (*ApoE*) KO mice with agonists reported to be selective for S1PR1 have been shown in some studies, but not others, to protect against atherosclerosis development [24,25,26]. Finally, selective endothelial cell-specific inactivation of the *S1pr1* gene in juvenile mice has been reported to increase atherosclerosis development in response to high fat (HF) diet feeding [27]. On the other hand, selective inactivation of the S1PR2 in bone marrow (BM)-derived cells has been reported to suppress atherosclerosis development in *ApoE* KO mice, due to suppression of inflammation [28]. The role of macrophage S1PR1 in atherosclerosis, however, has not been described.

In this study, we tested the role of S1PR1 expressed in macrophages in HF diet-induced atherosclerosis in *Ldlr* KO mice by first generating mice selectively lacking *S1pr1* gene expression in myeloid cells, including macrophages, and then transplanting BM from these mice into recipient *Ldlr* KO mice to generate chimeras that lacked *S1pr1* expression in BM-derived myeloid cells. We then initiated atherosclerosis development in these mice and control mice with normal *S1pr1* expression in BM derived cells by feeding them a HF diet. Selective inactivation of *S1pr1* in BM-derived myeloid cells accelerated the diet-induced development of atherosclerosis and necrotic cores within atherosclerotic plaques. We also found increased apoptosis in atherosclerotic plaques of HF-diet fed mice. We further show that, in cultured macrophages, the S1PR1 selective agonist SEW2871 and high density lipoprotein (HDL; reported to be a major plasma carrier of S1P, the natural ligand for S1PR1) were able to protect primary mouse macrophages from apoptosis, and that this involved SEW2871- or HDL-induced activation of the phosphatidylinositol-3-kinase (PI3K)/Akt signaling pathway. Together, these results demonstrate that S1PR1 in macrophages may be an important mediator of HDL dependent protection against cellular apoptosis and plays a role in delaying apoptosis and necrotic core development within atherosclerotic plaques.

## 2. Results

### 2.1. Selective Inactivation of S1PR1 in Myeloid Cells

**Figure 1:**
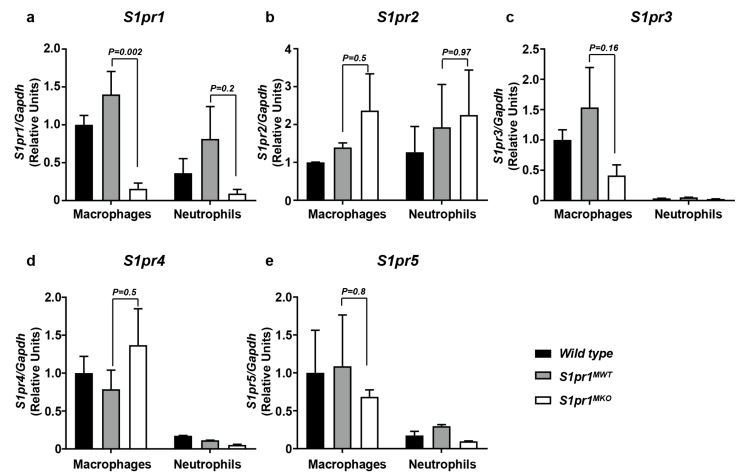
*S1pr1*, *2*, *3*, *4* and *5* gene expression in macrophages and neutrophils from *S1pr1^MKO^* mice. Thioglycollate-elicited peritoneal macrophages or neutrophils were harvested from wild-type (*WT*) C57BL6/J (black bars), *S1pr1^MWT^* (grey bars) and *S1pr1^MKO^* mice (white bars) and RNA extraction and quantitative real time, reverse transcriptase PCR was performed as described in the Methods section, for (**a**) *S1pr1*; (**b**) *S1pr2*; (**c**) *S1pr3*; (**d**) *S1pr4*; and (**e**) *S1pr5*. *Gapdh* was used as an internal control. Group sizes are (**a**): *n* = 5 for macrophages and *n* = 3 for neutrophils; (**b**): *n* = 4 for macrophages and *n* = 3 for neutrophils; (**c**): *n* = 6 for macrophages and *n* = 3 for neutrophils; (**d**,**e**): *n* = 3 for both macrophages and neutrophils, where each replicate represents cells isolated from a different mouse. Results are expressed as means ± SEM and are relative to levels detected in macrophages from *WT* mice (black bars). Data were analyzed by one-way ANOVA with Tukey’s multiple comparisons test. *p*-values are indicated on the graphs for comparisons between *S1pr1^MKO^* and *S1pr1^MWT^* cells. Gene expression levels in wild type and *S1pr1^MWT^* cells were not statistically significantly different (*p* > 0.3).

Myeloid-specific *S1pr1* KO (*S1pr1^MKO^*) mice were generated by mating *S1pr1^lox/lox^* mice, in which the *S1pr1* gene is flanked by LoxP recombination sites [29] with *Lys2^Cre/Cre^* mice, in which the bacterial Cre recombinase is knocked into the *Lys2* gene and expressed selectively in macrophages and granulocytes [30]. While generating the *S1pr1^MKO^* (i.e., *Lys2^Cre/Cre^S1pr1^lox/lox^*) mice from *Lys2^Cre/Cre^S1pr1^lox/wt^* parents, we observed that the overall proportion of double homozygous offspring recovered from multiple matings was only 53% of the expected Mendelian proportion; however, the *S1pr1^MKO^* mice themselves appeared healthy and produced offspring when mated (not shown). We tested the effects of the mutation on *S1pr1* expression in macrophages and in neutrophils, which are the most abundant granulocyte and have been shown to participate in atherogenesis [31,32]. Thioglycollate-elicited peritoneal macrophages and neutrophils were prepared from the resulting homozygous mutant *Lys2^Cre/Cre^ S1pr1^lox/lox^* mice (hereafter referred to as *S1pr1^MKO^* mice), and *Lys2^Cre/Cre^S1pr1^wt/wt^* control (hereafter referred to as *S1pr1^MWT^*) mice. As an additional control, we prepared thioglycollate-elicited peritoneal macrophages and neutrophils from wild type mice. Using quantitative real time RT-PCR, we found that S1PR1 transcript levels were not different between wild type or *S1pr1^MWT^* macrophages but were dramatically reduced in *S1pr1^MKO^* macrophages (Figure 1a). S1PR1 transcript levels in neutrophils from wild type or *S1pr1^MWT^* mice appeared to be lower than in macrophages from corresponding mice and we saw a trend towards reduced S1PR1 transcripts in neutrophils from *S1pr1^MKO^* compared to neutrophils from *S1pr1^MWT^* mice, which did not reach statistical significance (Figure 1a). We saw no statistically significant differences in the levels of S1PR2, 3, 4 or 5 in macrophages (Figure 1b–e), although there appeared to be a trend towards reduced S1PR3 in macrophages from *S1pr1^MKO^* compared to *S1pr1^MWT^* mice (Figure 1c). We saw no statistically significant differences in the levels of S1PR2 in neutrophils from *S1pr1^MKO^* compared to neutrophils from *S1pr1^MWT^* mice (Figure 1b). Levels of S1PR3, 4 and 5 were very low in neutrophils compared to macrophages (Figure 1c–e). This demonstrated that *S1pr1* expression was ablated in macrophages from *S1pr1^MKO^* mice and that there appeared to be no compensatory upregulation of *S1pr2* or *3* expression. 

### 2.2. Effect of Myeloid Selective S1PR1 Deficiency on HF Diet-Induced Atherosclerosis in BM Transplanted Ldlr KO Mice

**Figure 2:**
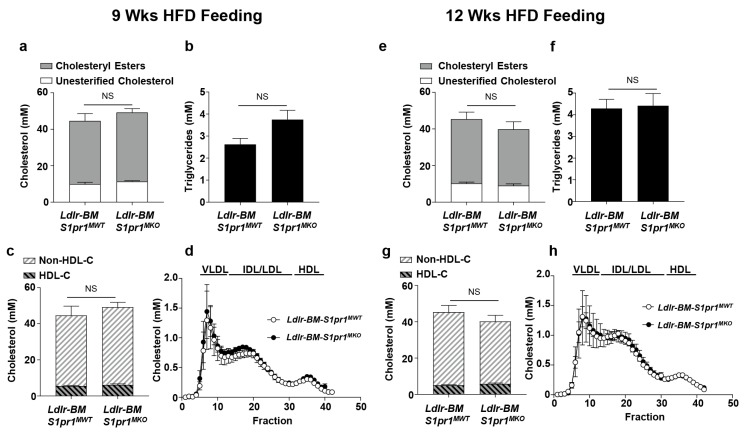
Plasma lipid and lipoprotein analysis in *Ldlr^BM S1pr1 MKO^* and *Ldlr^BM S1pr1 MWT^* mice fed a high fat diet (HFD) for nine or twelve weeks. *Ldlr^BM S1pr1 MKO^* and control *Ldlr^BM S1pr1 MWT^* generated by transplanting bone marrow (BM) from either *S1pr1^MKO^* or control *S1pr1^MWT^* mice into lethally irradiated *Ldlr* KO recipients as described in the Methods section. After four weeks of recovery from BM transplantation, chimeric mice were fed a HFD for (**a**–**d**) 9 weeks or (**e**–**h**) 12 weeks; (**a**,**e**) plasma levels of cholesterol ester (grey bar) and unesterified cholesterol (white bar); (**b**,**f**) triglycerides and (**c**,**g**) High density lipoprotein (HDL)-associated cholesterol (HDL-C) (black and grey striped bar) and non-HDL-C (white and grey striped bar). Group sizes are *n* = 13 *Ldlr^BM−S1PR1−MWT^* and *n* = 14 *Ldlr^BM S1pr1 MKO^* mice fed for 9 weeks and *n* = 19 *Ldlr^BM−S1PR1−MWT^* and *n* = 10 *Ldlr^BM S1pr1 MKO^* mice fed for 12 weeks; (**d**,**h**) average plasma lipoprotein total-cholesterol profiles for *n* = 3 *Ldlr^BM−S1PR1−MWT^* (white symbol) and *n* = 10 *Ldlr^BM S1pr1 MKO^* mice (black symbol) fed for either 9 or 12 weeks as indicated. Bars above the profiles indicate fractions in which purified human lipoproteins elute. Results are shown as means ± SEM. Data in panels b and f were analyzed by the Mann–Whitney rank sum test, while data in all other panels were analyzed by two-way ANOVA with Tukey’s multiple comparisons test. No statistically significant differences (NS) between *Ldlr^BM S1pr1 MKO^* and *Ldlr^BM S1PR1 MWT^* mice were found.

To test the role of S1PR1 in myeloid cells in atherosclerosis development, we transplanted BM from either *S1pr1^MKO^* or control *S1pr1^MWT^* mice into lethally irradiated *Ldlr* KO recipient mice (hereafter referred as *Ldlr^BM S1pr1 MKO^* and *Ldlr^BM S1pr1 MWT^* mice). Once the mice recovered from the BM transplantation procedure, atherosclerosis was induced by feeding the BM transplanted mice a HF-diet for either nine or twelve weeks. Atherosclerosis in HF-fed *Ldlr* KO mice is driven largely by hypercholesterolemia. Therefore, we first examined plasma and lipoprotein cholesterol levels in the HF-diet fed mice to determine if inactivation of *S1pr1* in BM-derived myeloid cells may have had unanticipated influences on steady state lipoprotein cholesterol levels. *Ldlr^BM S1pr1 MWT^* and *Ldlr^BM S1pr1 MKO^* mice fed the HF diet showed no significant differences in plasma or lipoprotein levels of cholesterol (total, unesterified, esterified, HDL or non-HDL) or triglycerides (Figure 2). 

**Figure 3:**
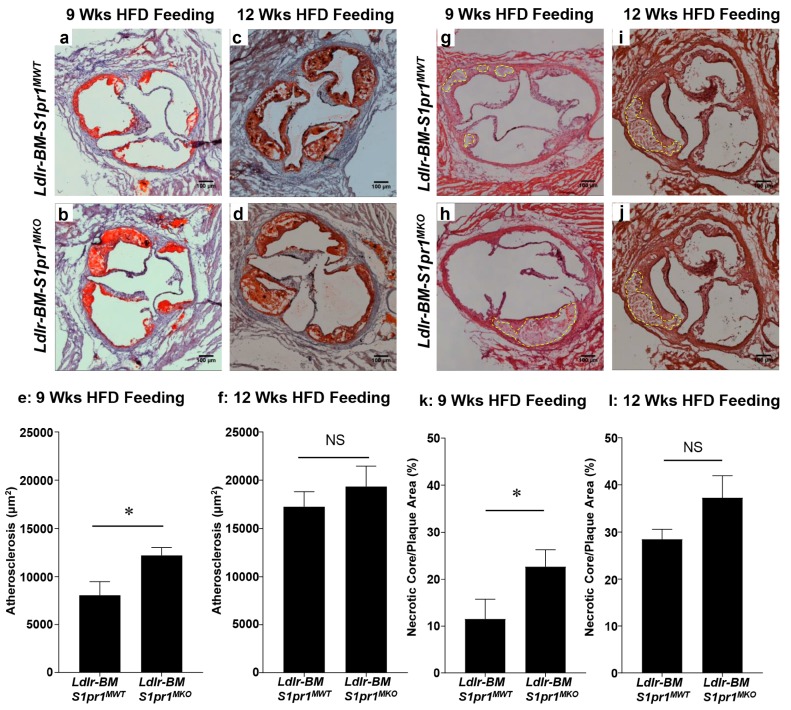
*S1pr1* deficiency in BM-derived myeloid cells in *Ldlr* KO mice accelerates HF-diet induced atherosclerosis and necrotic core formation. (**a**–**d**) Representative images of oil red O and hematoxylin stained atherosclerotic plaques in the aortic sinuses of *Ldlr* KO transplanted with BM from either *S1pr1^MKO^* or control *S1pr1^MWT^* donors and fed HF diet (HFD) for either nine or twelve weeks; (**e**,**f**) quantification of atherosclerotic plaque cross-sectional area for mice fed for nine weeks (panel (**e**)) and 12 weeks (panel (**f**)); (**g**–**j**) representative images of hematoxylin and eosin stained atherosclerotic plaque sections showing necrotic cores (outlined in yellow); (**k**,**l**) necrotic core areas were measured and normalized to the total atherosclerotic plaque cross-sectional areas in each section for mice fed the HFD for nine (panel (**k**)) and twelve weeks (panel (**l**)). Data are means ± SEM of *n* = 13 *Ldlr^−BM S1pr1 MWT^* and 14 *Ldlr^−BM S1pr1 MKO^* mice fed for nine weeks, and *n* = 19 *Ldlr^−BM S1pr1 MWT^* and 10 *Ldlr^−BM S1pr1 MKO^* mice fed for 12 weeks. Scale bars = 100 µm. Data were analyzed by the Mann–Whitney rank sum test; * indicates *p* < 0.05; NS (non-significant) indicates *p* > 0.05.

Despite this, atherosclerotic plaque cross-sectional areas were significantly increased in the aortic sinus of *Ldlr^BM S1pr1 MKO^* mice compared to control *Ldlr^BM S1pr1 MWT^* mice (122,000 ± 8000 μm^2^ versus 81,000 ± 14,000 μm^2^, respectively) after mice were fed the HF diet for nine weeks (Figure 3a,b,e). Average atherosclerotic plaque sizes in the aortic sinuses of both *Ldlr^BM S1pr1 MKO^* and control *Ldlr^BM S1pr1 MWT^* mice increased substantially (1.6–2.1-fold) after 12 weeks of HF diet-feeding (Figure 3c,d,f). Although there was a slight trend towards higher average plaque size in *Ldlr^BM S1pr1 MKO^* versus *Ldlr^BM S1pr1 MWT^* mice fed the HF diet for 12 weeks (193,000 ± 21,000 μm^2^ versus 173,000 ± 15,000 μm^2^, respectively), the data did not reach statistical significance.

**Figure 4:**
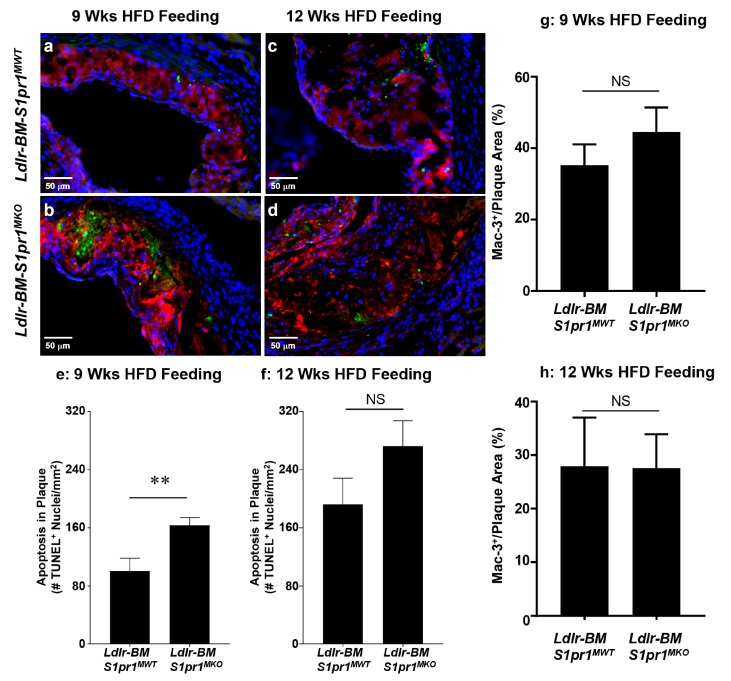
*S1pr1* deficiency in BM-derived myeloid cells accelerates accumulation of apoptotic cells in atherosclerotic plaques of HF-diet fed *Ldlr* KO mice. (**a**–**d**) representative images of atherosclerotic plaque sections stained for TUNEL + nuclei (green fluorescence), Mac-3 (macrophage marker, red fluorescence) and DAPI (nuclei, blue fluorescence). Scale bars represent 50 µm; (**e**,**f**) the numbers of TUNEL+ apoptotic cells within atherosclerotic plaques in each section were counted and normalized to the total atherosclerotic plaque cross-sectional areas in each section for mice fed the HF diet for nine (panel (**e**)) and twelve weeks (panel (**f**)). Data are means ± SEM of *n* = 10 *S1pr1^MWT^* and nine *S1pr1^MKO^* BM transplanted mice fed for nine weeks (panel (**e**)), and *n* = 9 *S1pr1^MWT^* and 10 *S1pr1^MKO^* BM transplanted mice fed for 12 weeks (panel (**f**)); (**g**,**h**) the cross-sectional area of Mac-3+ staining within plaques was normalized to the total atherosclerotic plaque area in the corresponding section and mean ± SEM values of *n* = 5 mice fed for nine weeks (panel (**g**)) and 12 weeks (panel (**h**)). Data were analyzed by the Mann–Whitney rank sum test; ** *p* = 0.01; NS (non-significant) indicates *p* > 0.05.

Atherosclerotic plaques from nine-week HF diet-fed *Ldlr^BM S1pr1 MKO^* mice exhibited larger sized necrotic cores (average 22.6 ± 3.6% of total plaque size) than similarly fed *Ldlr^BM S1pr1 MWT^* mice (average 11.5 ± 4.2% of total plaque size) (Figure 3g,h,k). As for average atherosclerotic plaque sizes, the relative necrotic core sizes increased 1.6–2.5-fold for both *Ldlr^BM S1pr1 MKO^* and control *Ldlr^BM S1pr1 MWT^* mice after 12 weeks of HF diet feeding (Figure 4i,j,l). Although there was a trend towards increased average necrotic core sizes in the *Ldlr^BM S1pr1 MKO^* compared to *Ldlr^BM S1pr1 MWT^* mice (37.3 ± 4.6 versus 28.4 ± 2.2% of total plaque size, respectively), the differences were not statistically significant after 12 weeks of HF-diet feeding. 

Terminal deoxynucleotidyl transferase dUTP nick end labeling (TUNEL) staining revealed significantly increased (163 ± 11 vs. 101 ± 17 /mm^2^ of atherosclerotic plaque) accumulation of apoptotic nuclei in plaques from *Ldlr^BM S1pr1 MKO^* mice compared to *Ldlr^BM S1pr1 MWT^* mice after nine weeks of HF diet feeding (Figure 4a,b,e). After 12 weeks of HF diet-feeding, apoptosis in atherosclerotic plaques increased 1.7–1.9-fold (Figure 4c,d,f) and there was a trend towards increased apoptotic nuclei in plaques from *Ldlr^BM S1pr1 MKO^* compared to *Ldlr^BM S1pr1 MWT^* mice (272 ± 35/mm^2^ versus 192 ± 36/mm^2^, respectively), but the differences were not statistically significant. Apoptotic nuclei were found in regions of atherosclerotic plaques that stained positively for the Mac-3 macrophage marker (Figure 4a–d). No differences were observed in the extent of Mac-3 staining in atherosclerotic plaques from *Ldlr^BM S1pr1 MKO^* compared to *Ldlr^BM S1pr1 MWT^* mice (Figure 4g,h). Together, these data demonstrate that inactivation of S1PR1 in BM-derived myeloid cells accelerates HF diet-induced development of atherosclerosis, necrotic cores and apoptosis within atherosclerotic plaques of *Ldlr* KO mice, exhibiting a more pronounced effect at earlier stages of HF diet feeding.

### 2.3. SEW2871, a Selective S1PR1 Agonist, Protects Macrophages against Apoptosis

**Figure 5:**
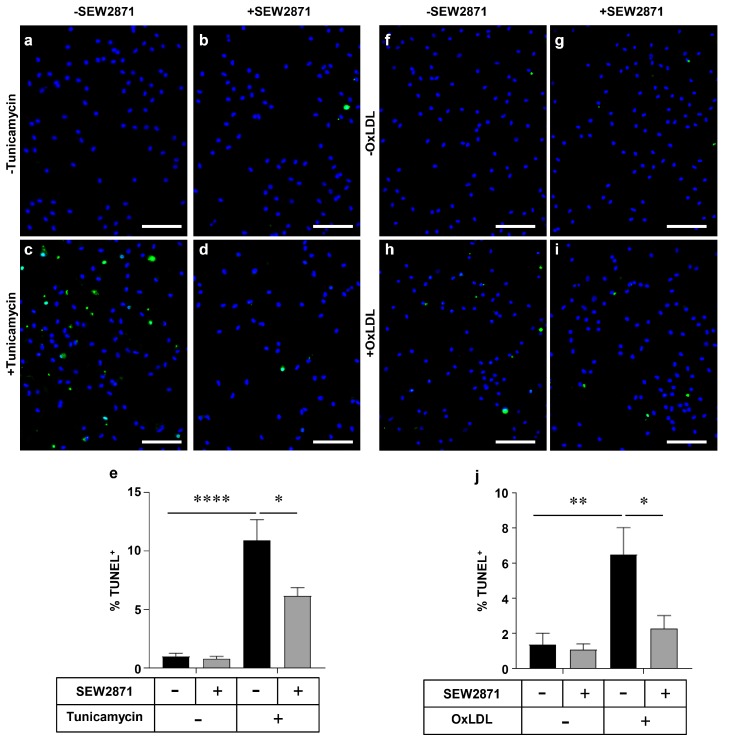
The S1PR1 selective agonist SEW2871 protects macrophages against tunicamycin- and oxLDL-induced apoptosis. Thioglycollate-elicited peritoneal macrophages collected from wild type (*WT*) mice were treated in culture with either tunicamycin (10 µg/mL) or oxLDL (100 µg protein/mL) in the presence of 1 µm SEW2871 added as a 500× stock in DMSO. Control cells lacking SEW2871 treatment were treated with an equivalent volume of DMSO solvent. After 24 h, cells were fixed and apoptosis was detected by TUNEL staining (green fluorescence) as described in the Methods section. Nuclei were stained using DAPI (blue). (**a**–**d**) Representative images of TUNEL/DAPI-stained macrophages for tunicamycin-treated and control cells incubated with or without SEW2871; scale bars represent 50 µm. (**e**) Quantification of the % of TUNEL^+^ nuclei from tunicamycin-, SEW2871- and control-treated samples as a measure of the extent of apoptosis. *n* = 6, where each replicate represents cells prepared from a different mouse. (**f**–**i**) Representative images of TUNEL/DAPI-stained macrophages for oxLDL-treated and control cells incubated with or without SEW2871; scale bars represent 50 µm. (**j**) Quantification of the % of TUNEL^+^ nuclei from oxLDL-, SEW2871- and control-treated samples as a measure of the extent of apoptosis. *n* = 5, where each replicate represents cells prepared from a different mouse. Results are shown as means ± SEM. Data were analyzed by two-way ANOVA with Tukey’s multiple comparisons test; * *p* < 0.05, ** *p* < 0.01, **** *p* < 0.0001.

To explore the mechanisms by which inactivation of S1PR1 expression in BM-derived myeloid cells, including macrophages, accelerated the accumulation of apoptotic nuclei in atherosclerotic plaques of BM transplanted and HF diet-fed *Ldlr* KO mice, we examined the effects of treatment of primary mouse macrophages with SEW2871, a selective S1PR1 agonist [33], on apoptosis induced by either tunicamycin, an inhibitor of protein glycosylation that induces apoptosis secondary to ER-stress, and oxLDL, an inducer of oxidative stress [9,10,17,18]. No evidence of apoptosis above baseline levels was detected in macrophages treated with SEW2871 alone (Figure 5a,b,f,g). When macrophages were exposed to 10 µg/mL tunicamycin, a significant increase in apoptosis was detected (Figure 5a,c,e). Cells co-incubated with tunicamycin in presence of 1 µm SEW2871 exhibited significantly (~40%) reduced levels of apoptosis compared to macrophages treated with tunicamycin alone (Figure 5c–e). Similar results were observed when apoptosis was triggered by exposure of macrophages to oxLDL (100 µg protein/mL). OxLDL-treated macrophages exhibited significantly increased apoptosis compared to untreated macrophages (Figure 5f,h,j), while treatment with SEW2871 reduced the level of oxLDL-induced apoptosis by approximately 65% (Figure 5h–j). Together, these results suggest that the S1PR1 selective agonist, SEW2871, protects macrophages against apoptosis induced by either the ER stress-inducing agent tunicamycin or by oxLDL. 

### 2.4. Role of the PI3K/Akt Pathway in S1PR1 Agonist-Mediated Protection against Macrophage Apoptosis

**Figure 6:**
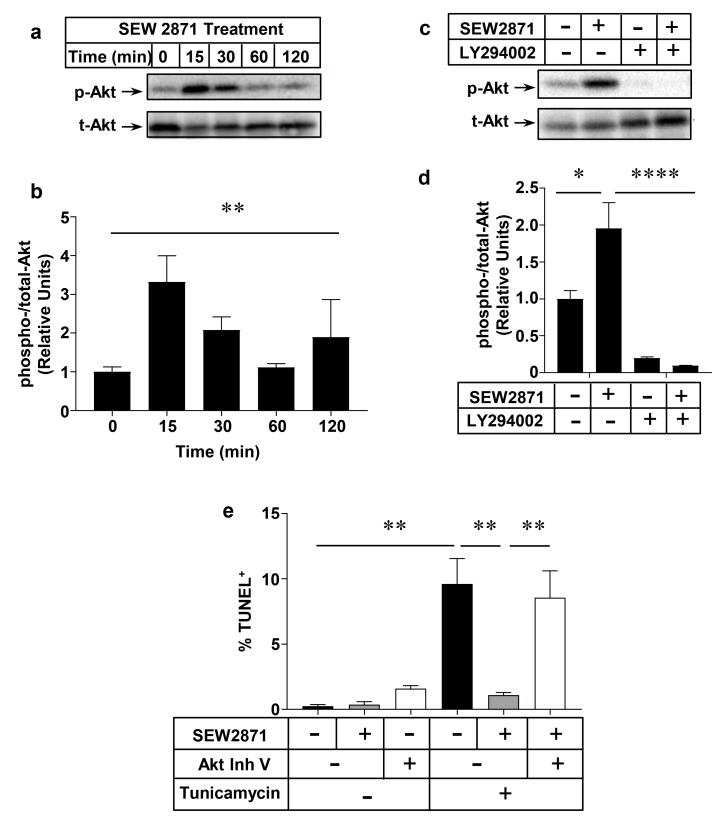
PI3K/Akt activation is required for SEW2871-mediated protection of macrophages against apoptosis. Thioglycollate-elicited peritoneal macrophages collected from wild type (*WT*) mice were treated in culture as indicated. (**a**,**b**) cells were incubated with 1 µm SEW2871 for the indicated periods of time. Cells were lysed and extracts prepared and analyzed by immunoblotting for phospho Ser 473 Akt (p-Akt) or total-Akt (t-Akt). (**a**) representative immunoblot; (**b**) quantification of the relative band intensities for phospo-Akt:total-Akt at each time point. Data are means ± SEM of *n* = 3 samples, where each represents cells prepared from a different mouse; (**c**) representative immunoblot of p-Akt and t-Akt; and (**d**) quantification of relative levels of phospho-Akt/total-Akt for cells (from *n* = 4 mice) treated in culture without or with 1 μm SEW2871 (S1PR1 selective agonist), 10 µm LY294002 (PI3K inhibitor), neither or both as indicated, for 15 min; (**e**) quantification of the % TUNEL^+^ nuclei as a measure of apoptosis in each sample after treatment of cells for 24 h with 10 µg/mL tunicamycin, 1 µm SEW2871, 10 µm Akt inhibitor V (Akt Inh. V) or combinations as indicated. Data are means ± SEM of *n* = 3 replicates, where each replicate represents cells prepared from a different mouse. Data in (**b**,**e**) were analyzed by 1-way ANOVA with Tukey’s multiple comparisons test; data in (**d**) were analyzed by two-way ANOVA with Tukey’s multiple comparisons test; * *p* < 0.05; ** *p* < 0.01; **** *p* < 0.0001.

We have previously reported that PI3K and Akt1 signaling is involved in SEW2871 induced macrophage migration [34]. To test the involvement of Akt in SEW2871 mediated protection of macrophages against apoptosis, peritoneal macrophages from wild type, C57BL6/J mice were incubated with 1 µm SEW2871 for different periods of time, from 15 min to 2 h. Untreated macrophages were included as a 0 time control. Immunoblotting for Akt phosphorylated at Ser 473 (p-Akt) and total Akt levels (Figure 6a,b) revealed that SEW2871-treatment of peritoneal macrophages from wild type mice resulted in induction of Akt phosphorylation with levels peaking at 15 min and declining thereafter. The transient increase in p-Akt after 15 min of incubation with SEW2871 was blocked by pre-treatment of cells with the PI3K inhibitor LY294002 (Figure 6c,d). To test if Akt is directly involved in SEW2871-mediated protection against apoptosis, macrophages were incubated with a pan-Akt inhibitor (Akt Inh. V) before exposure to tunicamycin and SEW2871. Akt Inh. V-treatment, alone, appeared to slightly increase the baseline level of apoptosis although this did not reach statistical significance (Figure 6e). As above, tunicamycin treatment induced macrophage apoptosis and treatment with SEW2871 protected them (in this experiment, fully) against tunicamycin-induced apoptosis (Figure 6e). On the other hand, macrophages that were pre-treated with Akt Inh. V were not protected by SEW2817 against tunicamycin-induced apoptosis (Figure 6e). Together these results demonstrate that the S1PR1 selective agonist SEW2871 protects macrophages from apoptosis through activation of the PI3K/Akt signaling pathway.

### 2.5. Effect of Macrophage S1PR1 Deficiency on Protection against Apoptosis

**Figure 7:**
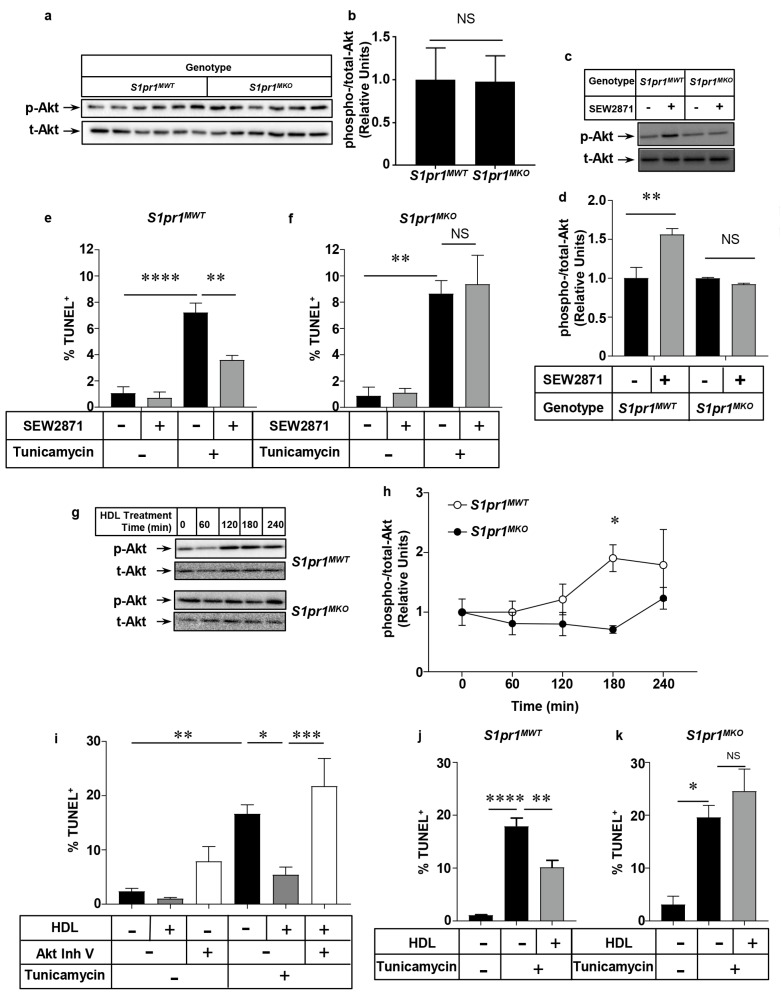
Inactivation of *S1pr1* expression in macrophages prevents SEW2871- or HDL-stimulated Akt phosphorylation and protection against tunicamycin-induced apoptosis. Thioglycollate-elicited peritoneal macrophages, prepared from *S1pr1^MKO^* and control *S1pr1^MWT^* mice were either untreated or treated in culture with 1 µm SEW2871, 50 µg (protein)/mL HDL, 10 µg/mL tunicamycin, or combinations as indicated. (**a**) representative immunoblotting for phospho-Akt (p-Akt) and total-Akt (t-Akt) in lysates from unstimulated *S1pr1^MKO^* and control *S1pr1^MWT^* macrophages; (**b**) quantification of the relative band intensities for phospo-Akt:total-Akt in unstimulated cells. Data are means ± SEM of *n* = 6 samples where each represents cells prepared from a different mouse; (**c**) representative immunoblotting for phospho-Akt (p-Akt) and total-Akt (t-Akt) in lysates prepared after 15 min of treatment without or with SEW2871; (**d**) quantification of the relative band intensities for phospo-Akt:total-Akt in cells treated with SEW2871 for 15 min. Data are means ± SEM of *n* = 3 samples, where each represents cells prepared from a different mouse; (**e**,**f**) quantification of the% TUNEL^+^ nuclei as a measure of apoptosis after treatment of control *S1pr1^MWT^* (**e**) or *S1pr1^MKO^* macrophages (**f**) for 24 h with tunicamycin, SEW2871 or combinations as indicated. Data are means ± SEM of *n* = 3 replicates, where each replicate represents cells prepared from a different mouse (**g**) representative immunoblots for phospho-Akt (p-Akt) and total-Akt (t-Akt); and (**h**) quantification of relative band intensities (relative to 0 time) of phospho-Akt/total-Akt in *S1pr1^MWT^* and *S1pr1^MKO^* macrophages incubated with HDL for the indicated times; (**i**) apoptosis (% TUNEL^+^ nuclei) after 24 h treatment of macrophages from wild type mice with combinations of tunicamycin, HDL and the pan-Akt inhibitor Akt Inh. V as indicated; (**j**,**k**) apoptosis (% TUNEL^+^ nuclei) after treatment of control (**j**) *S1pr1^MWT^* or (**k**) *S1pr1^MKO^* macrophages for 24 h with tunicamycin or HDL (at the concentrations indicated above). Data are means ± SEM. Data in panel (**a**) are *n* = 6 replicates, and panels (**d**–**f**,**h**,**j**) and (**k**) are *n* = 3 replicates, where each replicate represents cells prepared from a different mouse. Data in panel (**i**) were from *n* = 11–12 replicates comprised of cells prepared from three different mice. Data in (**b**) were analyzed by the Mann–Whitney Rank Sum test. Data in d and h were analyzed by two-way ANOVA with Tukey’s multiple comparisons test. Data in (**e**,**f**,**i**,**j**,**k**) were analyzed by one-way ANOVA with Tukey’s multiple comparisons test; * *p* < 0.05, ** *p* < 0.01; *** *p* < 0.001; **** *p* = 0.0001. NS (non-significant) indicates *p* > 0.05.

To directly test the involvement of S1PR1 in SEW2871-mediated activation of Akt and protection of macrophages against apoptosis, we prepared thioglycollate-elicited peritoneal macrophages from *S1pr1^MKO^* mice (which lack S1PR1 expression in macrophages; Figure 1a) and control *S1pr1^MWT^* mice (which have normal levels of *S1pr1* gene expression in macrophages; Figure 1a) and tested their responses to SEW2871 in culture. Untreated macrophages from control *S1pr1^MWT^* and *S1pr1^MKO^* mice had similar baseline p-Akt levels (Figure 7a,b). SEW2871-treatment for 15 min robustly increased p-Akt levels in control *S1pr1^MWT^* macrophages, but failed to induce elevated p-Akt levels in *S1pr1^MKO^* macrophages in which S1PR1 was knocked out (Figure 7c,d). Furthermore, SEW2871 treatment suppressed tunicamycin-induced apoptosis in macrophages from control *S1pr1^MWT^* mice (Figure 7e), similar to effects seen in macrophages from wild type, C57BL6/J mice (Figure 5e), but failed to suppress tunicamycin-induced apoptosis in macrophages from *S1pr1^MKO^* mice, lacking *S1pr1* expression (Figure 7f), even though levels of tunicamycin-induced apoptosis in the absence of SEW2871 were similar between the macrophages from *S1pr1^MKO^* and *S1pr1^MWT^* mice. 

### 2.6. HDL Protects Macrophages from Apoptosis in an Akt and S1PR1 Dependent Manner

**Figure 8:**
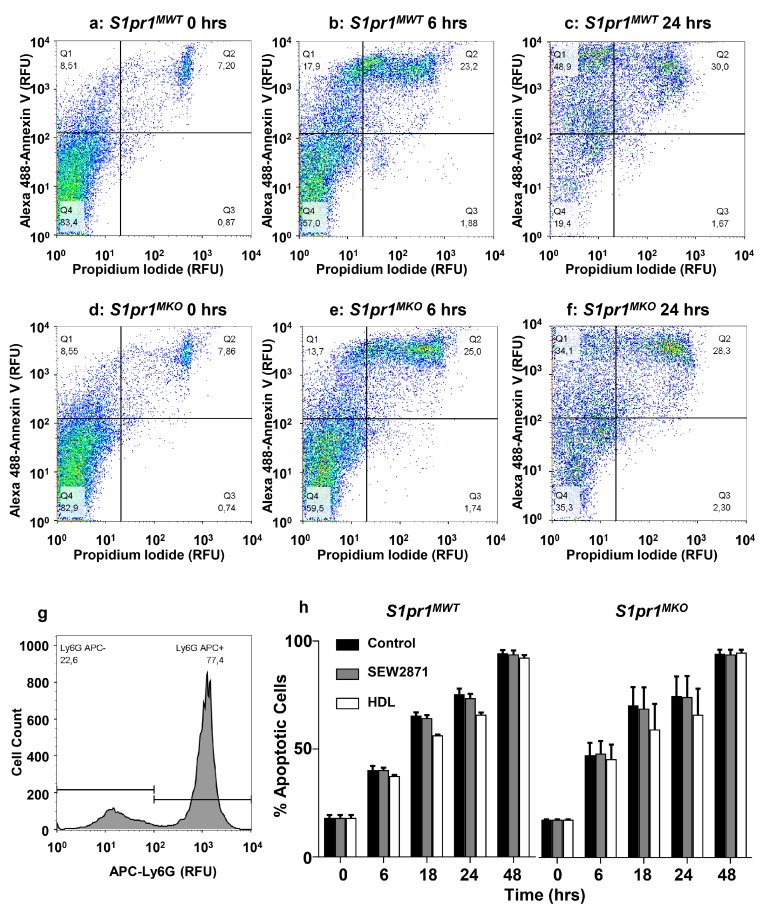
Apoptosis of neutrophils from *S1pr1^MKO^* or *S1pr1^MWT^* mice is unaffected by HDL or SEW2871 treatment. Neutrophils prepared from *S1pr1^MKO^* or *S1pr1^MWT^* mice were either not treated or treated with 1 µm SEW2871 or 50 μg (protein)/mL HDL and cultured for up to 48 h. At different times, cells were washed, stained with APC-labeled Ly6G (neutrophil marker), Alexa 488-labeled annexin V and propidium iodide and analyzed by flow cytometry. (**a**–**f**) representative scatter plots of Alexa 488-annexin V and propidium iodide staining of untreated neutrophils from (**a**–**c**) *S1pr1^MWT^* mice or (**d**–**f**) *S1pr1^MKO^* mice after (**a**,**d**) 0, (**b**,**e**) 6 and (**c**,**f**) 24 h in culture; (**g**) representative histogram of APC-Ly6G staining, showing the Ly6G-positive population of cells analyzed for annexin V and propidium iodide staining; (**h**) quantification of apoptosis (% cells that were positive for Alexa 488-annexin V staining) in cultures of neutrophils from *S1pr1^MWT^* or *S1pr1^MKO^* mice over 48 h in culture without or with SEW2871 or HDL treatment. Data in h are means ± SD of *n* = 3 replicates where each replicate represents cells prepared from a different mouse. Data were analyzed by two-way repeated measures ANOVA. Apoptosis significantly increased over time (*p* < 0.0001) but values for *S1pr1^MWT^* or *S1pr1^MKO^* neutrophils or treatment with HDL or SEW2871 were not statistically significantly different (*p* > 0.3).

S1P, the natural ligand of S1PR1, is reportedly mainly associated with HDL in plasma in both mice and humans and HDL-associated S1P has been reported to mediate some of HDL’s beneficial effects on cells [35] (reviewed in [36]). We therefore tested the ability of HDL to induce Akt phosphorylation and protection against tunicamycin-induced apoptosis in order to examine the role of macrophage S1PR1 in this process. Treatment of macrophages from *S1pr1^MWT^* mice with HDL isolated from human plasma resulted in a time-dependent increase in Akt phosphorylation (Figure 7g, upper panel, and Figure 7h). The time-course of HDL-induction of p-Akt (peaking at 3 h) was much slower than that induced by treatment with SEW2871 (peaking at 15 min—see Figure 6a,b). In contrast, HDL treatment of *S1pr1^MKO^* macrophages did not significantly increase p-Akt levels indicating that S1PR1 was required for HDL’s ability to induce activation of Akt in mouse peritoneal macrophages (Figure 7g, lower panel, and Figure 7h). HDL was able to suppress (by 68%) tunicamycin-induced apoptosis in macrophages from wild type mice in a manner that was attenuated by pre-treatment of macrophages with the Akt Inh. V (Figure 7i), demonstrating that HDL-mediated protection against tunicamycin-induced apoptosis required Akt activation. In a separate experiment with a different batch of HDL, tunicamycin-induced apoptosis was reduced by about 43% upon HDL treatment of *S1pr1^MWT^* macrophages (Figure 7j), whereas HDL-treatment did not suppress tunicamycin-induced apoptosis in macrophages from *S1pr1^MKO^* mice, which lacked *S1pr1* gene expression (Figure 7k). In contrast, we saw no differences in the extent of apoptosis in neutrophils prepared from *S1pr1^MKO^* versus *S1pr1^MWT^* mice, either in the absence of treatment or after treatment with SEW2871 or HDL at concentrations that attenuated apoptosis in macrophages (Figure 8). These findings demonstrate that S1PR1 is responsible for the ability of the HDL, an endogenous suppressor of macrophage apoptosis, to induce Akt activation and suppress apoptosis in mouse macrophages. This provides a likely explanation for the accelerated accumulation of apoptotic cells in atherosclerotic plaques observed in *Ldlr* KO mice transplanted with BM from *S1pr1^MKO^* versus *S1pr1^MWT^* mice (Figure 4g–i). 

## 3. Discussion

Previous studies have reported that attenuating S1P signaling increases atherosclerosis, while enhancing S1P signaling protects against atherosclerosis in mouse models. Specifically, lowering S1P concentrations through inhibition of sphingosine kinase 1 was reported to enhance high cholesterol diet-induced atherosclerosis in *Ldlr* KO mice [23] while raising S1P levels through inactivation of S1P lyase in BM-derived cells of *Ldlr* KO mice reduced HF diet-induced atherosclerosis [22]. Similarly, overexpression of apoM, the apolipoprotein carrier of S1P on HDL, has been reported to reduce diet induced atherosclerosis in *Ldlr* KO mice [37]. Treatment of mice with FTY720, a pro-agonist of multiple S1P receptors, including S1PR1, or with KRP203, a pro-agonist of S1PR1 and S1PR4 [33] has been reported in some studies to reduce atherosclerosis development in *Ldlr* KO mice induced by high cholesterol diet feeding [24,26]. On the other hand, another study reported that neither FTY720-treatment nor treatment with an S1PR1 selective agonist, CYM5442, reduced atherosclerosis in HF diet-fed *Ldlr* KO mice, the lack of effect being attributed to the low cholesterol content of the diet [25]. The studies reporting atheroprotective effects of either raising S1P levels or treating mice with FTY720 or KRP203 reported corresponding reductions in levels of circulating CD4^+^ and/or CD8^+^ T lymphocytes, suggesting that anti-inflammatory effects of these manipulations may have contributed to atheroprotection [24,25,26]. FTY720 indeed is anti-inflammatory, resulting in low levels of circulating lymphocytes. It is known to act as a functional antagonist of S1PR1, attenuating cell surface S1PR1 levels and signaling due to internalization of S1PR1 with prolonged exposure, an effect previously reported to underlie FTY720-mediated prevention of lymphocyte egress from lymphoid tissues into circulation [19,38]. As a result, it has not been possible to examine the role of S1PR1 in macrophages within atherosclerotic plaques in those studies. Hla and co-workers reported that selective endothelial cell specific *S1pr1* KO induced in juvenile *ApoE* KO mice resulted in enhanced HF diet-induced atherosclerosis [27]. To examine the influence of macrophage S1PR1 on atherosclerosis, we generated myeloid specific *S1pr1* KO mice and used these as donors in BM transplantation studies to generate *Ldlr* KO chimeras, which either selectively lack or have normal levels’ *S1pr1* gene expression in BM-derived myeloid cells. We demonstrate that selective *S1pr1* KO in BM-derived myeloid cells accelerates the high-fat diet-induced development of atherosclerosis in *Ldlr* KO mice. Atherosclerotic plaque sizes and relative necrotic core sizes were both increased by BM-selective, myeloid-specific *S1pr1* deficiency in *Ldlr* KO mice. Macrophage cell death is a major contributor to the development of large necrotic cores in atherosclerotic plaques [5,6]. Consistent with the increased necrotic core sizes seen in plaques from *Ldlr* KO mice lacking S1PR1 in BM-derived myeloid cells, these mice also exhibited higher levels of cellular apoptosis within atherosclerotic plaques than *Ldlr* KO mice with intact myeloid S1PR1 after HF diet-feeding for nine weeks. Although we saw trends towards increased atherosclerotic plaque sizes, necrotic core sizes and plaque apoptosis in *Ldlr* KO mice transplanted with BM from *S1pr1^MKO^* compared to control *S1pr1^MWT^* donors after 12 weeks of HF-diet feeding, the results did not reach statistical significance. This suggests that selective inactivation of *S1pr1* in BM-derived myeloid cells accelerated the HF-diet induced development of atherosclerotic plaques, necrotic cores and accumulation of apoptotic cells within plaques in *Ldlr* KO mice. 

We demonstrated in primary macrophages that the S1PR1-selective agonist SEW2871 was able to induce the activation of Akt phosphorylation and inhibit apoptosis induced by either the ER stressor, tunicamycin, or by oxLDL. ER stress in macrophages is activated at all stages of atherosclerosis development and oxLDL is present and thought to be a major driver of atherosclerosis in both humans and mouse models [9,10,12,13,17,18]. Our data demonstrates that PI3K/Akt signaling is required for SEW2871 mediated protection against tunicamycin-induced apoptosis in macrophages. We also demonstrate that the effects of SEW2871 on Akt activation and protection against apoptosis are entirely dependent on S1PR1 since they are lost when the *S1pr1* gene is inactivated in macrophages. We also demonstrate that HDL induced the phosphorylation of Akt in macrophages and protected them against tunicamycin-induced apoptosis in a manner that was sensitive to Akt inhibition. Again, this was dependent on S1PR1 since HDL neither induced Akt phosphorylation nor protected against tunicamycin-induced apoptosis in macrophages that were deficient in *S1pr1* gene expression. The majority of S1P, the natural ligand for S1PR1, is reportedly carried in plasma associated with HDL and many of HDL’s atheroprotective effects on vascular cells have been attributed to S1P. For example, it has been reported that HDL-associated S1P preferentially stimulates S1PR1 in endothelial cells leading to suppression of inflammatory activation of vascular cell adhesion molecule-1 and intercellular adhesion molecule-1, factors responsible for monocyte recruitment and the initiation of atherosclerosis [27,36,39,40,41]. Our data suggests that HDL activates Akt via the S1PR1; we think this is likely due to HDL associated S1P. However, we noted that the time-course of HDL stimulated Akt phosphorylation (2–3 h) was slower than that stimulated by the S1PR1 agonist SEW2871 (peak Akt phosphorylation at 15 min). This longer time-course may reflect a requirement for the transfer of S1P from HDL and delivery to S1PR1 for signaling, a process likely circumvented by direct S1PR1 activation by SEW2871. We hypothesize that this transfer could be mediated by the scavenger receptor class B, type 1 (SR-B1), an HDL receptor known to transport diverse lipids from bound HDL into cells [42]. Indeed, many reports, including our own, have demonstrated a key role for SR-B1 in HDL-mediated activation of Akt in different cell types including macrophages [34,43,44,45,46,47,48]. Further research is required to determine if the difference in the time-course of Akt activation by HDL versus SEW2871 reflects SR-B1 mediated uptake of S1P from bound HDL and delivery to S1PR1, versus direct activation of S1PR1 by SEW2871 via a pathway that does not require lipid transfer by SR-B1.

We have previously reported that HDL and agonists of S1PR1 each stimulate macrophage chemotaxis, and that an S1PR1-selective antagonist was able to block this effect of HDL, implicating S1PR1 in HDL dependent stimulation of macrophage migration [34]. A recent paper reported that HDL protects tumor-derived, immortal murine RAW264.7 and human THP-1 macrophage-like cells from apoptosis induced by treatment with etoposide or a combination of thapsigargin and fucoidan, in a manner dependent on HDL-associated S1P and cellular signal transducer and activator of transcription 3 (STAT3) and Janus Kinase 2 (JAK2) [49]. This study reported that selective agonists of S1PR1 were able to protect against etoposide or thapsigargin/fucoidan-induced apoptosis in RAW264.7 and THP-1 derived macrophages in a manner similar to HDL and to S1P, but that S1PR1-specific antagonists did not inhibit the protective effects of HDL. Instead, the protective effects of HDL were inhibited by antagonists selective for S1PR2 or S1PR3, suggesting that S1PR2 and 3 but not S1PR1 mediate HDL-dependent protection of macrophages from apoptosis [49]. Those findings, therefore, differ from ours, which demonstrate that S1PR1 plays an important role in protection against macrophage apoptosis in vitro and in vivo, although we cannot rule out the possibility that other S1P receptors may also be involved. For example, we did observe what appeared to be a trend towards reduced *S1pr3* expression in macrophages from *S1pr1^MKO^* mice, although this did not reach statistical significance. Furthermore, we cannot rule out the possibility that increased accumulation of apoptotic cells in atherosclerotic plaques observed in the nine-week fed *Ldlr^BM−S1pr1−MKO^* mice compared to the *Ldlr^BM−S1pr1−MWT^* mice may reflect reduced clearance of apoptotic cells. In this respect, others have reported that S1PR1 signaling plays a role in apoptotic cell clearance by macrophages [50]. Irrespective of this, our data clearly demonstrates that S1PR1 signaling protects cultured macrophages from apoptosis. 

Our findings also point to an important role played by the PI3K/Akt signaling pathway in S1PR1- and HDL-dependent signaling leading to protection against apoptosis. This is consistent with other reports that the PI3K/Akt pathway is activated downstream of S1PR1 and/or HDL signaling in macrophages and other cell types, including endothelial cells [19,34,43,44,45,46,47,48,51,52]. In endothelial cells, both HDL and S1PR1 signaling have been shown to lead to activation of endothelial NO synthase downstream of Akt activation [46,51]. The PI3K/Akt pathway has been demonstrated to play an important role in both inhibition of apoptosis and activation of cell survival pathways [53]. Several studies [54,55,56,57] have suggested reciprocal cross-talk between the PI3K/Akt pathway, implicated here in HDL dependent protection, and the JAK2/STAT3 signaling pathway implicated by others in HDL/S1P mediated protection of macrophages from apoptosis [49]. Further studies are required to determine the relationship between the PI3K/Akt and JAK2/STAT3 signaling pathways and their relative roles in S1PR1-dependent protection against apoptosis in macrophages. 

## 4. Materials and Methods

### 4.1. Materials

SEW2871 and tunicamycin were purchased from Cayman Chemicals (Ann Arbor, MI, USA). OxLDL and HDL were purchased from Alfa Aesar (Ward Hill, MA, USA). LY294002 (PI3K inhibitor) was purchased from Biovision Inc. (Milpitas, CA, USA). Akt inh. V (triciribine) was purchased from Millipore Corporation Canada (Etobicoke, ON, Canada). All other chemicals were purchased from Sigma Aldrich (St. Louis, MO, USA) unless indicated otherwise.

### 4.2. Mice

All procedures involving animals were approved by the McMaster University Animal Research Ethics Board (Animal Utilization Protocols 13 March 2011: 31 March 2011–31 March 2015; and 7 March 2015: 31 March 2015–31 March 2019) and were in accordance with guidelines of the Canadian Council of Animal Care. All animals were bred and housed in vented cages at the Thrombosis and Atherosclerosis Research Institute animal facility under controlled light (12 h light/dark cycle) and temperature conditions, and had free access to normal chow diet (Harlan Teklad TD2918, Madison, WI, USA) and automated watering. All mice were on a C57BL6/J background. *Ldlr* KO, wild type C57BL/6 and *Lys2-Cre* mice (in which Cre recombinase is knocked in to the *Lys2* gene, inactivating it and resulting in Cre expression in macrophages and granulocytes [30]) were each bred from founders obtained from the Jackson Laboratories (Bar Harbor, ME, USA). *S1pr1*-floxed mice were generously provided by Professor Richard Proia (National Institute of Diabetes and Digestive and Kidney Diseases, Bethesda, MD, USA). 

### 4.3. BM Transplantation

BM was flushed out of femurs and tibias from female mice with Iscove’s Modified Dulbecco’s media (Gibco, Thermo Fisher Scientific, Ottawa, ON, Canada) containing 2% heat inactivated FBS, and supplemented with 2 mM l-glutamine, 50 µg/mL penicillin and 50 U/mL streptomycin. Recipient mice (*Ldlr* KO, 10–12 weeks old) were exposed to 1300 rad of ^137^Cs irradiation using a Gammacell 3000 small animal irradiator (Best Theratronics, Ottawa, ON, Canada). BM (3 × 10^6^ cells/mouse) was injected intravenously via the tail vein. Mice were allowed to recover for 4 weeks, after which, atherosclerosis was induced by feeding a HF diet containing 21% butter fat and 0.15% cholesterol (catalogue number 112,286; Dyets Inc., Bethlehem, PA, USA) for 9 or 12 weeks. At the end of the feeding period, mice were fasted for 4 h prior to isoflurane anesthesia and euthanasia. Heparinized blood was collected by cardiac puncture and plasma was obtained by centrifugation at 4000 rpm in a microcentrifuge. Tissues were collected after in situ perfusion with 10 U/mL heparinized saline followed by 10% formalin, immersion fixed overnight in 10% formalin, and embedded in Shandon Cryomatrix (Thermo Fisher Scientific, Ottawa, ON, Canada). Plasma and tissues were stored at −80 °C until further analysis.

### 4.4. Plasma Lipids 

Total cholesterol, free cholesterol, triglycerides and HDL cholesterol in plasma were measured using enzymatic assay kits according to the manufacturer’s protocols (total cholesterol: Infinity Cholesterol, Thermo Fisher Scientific, Ottawa, ON, Canada; free cholesterol: Free Cholesterol E, Wako Diagnostics, Mountain View, CA, USA; triglycerides: l-type triglyceride M, Wako Chemicals, Richmond, VA, USA; HDL cholesterol: HDL-cholesterol E, Wako diagnostics, Mountain View, CA, USA). Non-HDL cholesterol was calculated as the difference between total cholesterol and HDL cholesterol measurements. Cholesteryl ester levels were calculated as the difference between total cholesterol and free cholesterol measurements for each sample. For lipoprotein total cholesterol profiles, plasma was separated by size by gel filtration chromatography using a Tricorn Superose 6 HR 10/300 column on an AKTA fast protein liquid chromatography system (GE Healthcare Life Sciences, Mississauga, ON, Canada), and total cholesterol was measured in each fraction as previously described [58,59]. 

### 4.5. Histology

Transverse cryosections (10 µm) of the aortic sinus were collected and stained with oil red O for neutral lipids and hematoxylin for nuclei. For detection of necrotic cores, aortic sinus cryosections were stained with hematoxylin/eosin (H & E). Necrotic cores were defined as the a-cellular, a-nuclear areas within the plaque. All images were collected using a Zeiss Axiovert 200 M inverted microscope using bright-field illumination (Carl Zeiss Canada Ltd., Toronto, ON, Canada). Necrotic core sizes and total plaque areas were measured as previously described [60] using quantitative morphometry with Image J software (Version 1.43m, Wayne Rasband, National Institutes of Health, Bethesda, MD, USA). Necrotic core sizes were normalized to total plaque areas for each sample. 

For detection of apoptosis in atherosclerotic plaques, 10 µm cryosections of the aortic sinus were stained using the ApopTag-Fluorescein In Situ Apoptosis detection kit (EMD Millipore, Etobicoke, ON, Canada) and nuclei were counterstained with DAPI as above. Macrophages within the aortic sinus were detected with immunofluorescence by staining with rat anti-mouse Mac-3 (CD107b) antibody (BD Biosciences, Mississauga, ON, Canada) followed by staining with goat anti-rat Alexa Fluor 594 conjugated IgG antibody (Invitrogen, Burlington, ON, Canada). All images were captured using a Zeiss Axiovert 200 M inverted fluorescence microscope with a 20× objective. TUNEL^+^ cells within the atherosclerotic plaque were counted and normalized to the atherosclerotic plaque area. Total area of Mac-3 staining within the atherosclerotic plaques was normalized to atherosclerotic plaque area.

### 4.6. Cell Preparation, Culture and Treatment

Peritoneal macrophages were elicited by intraperitoneal injection of 1 mL of 10% thioglycollate. At day 4 post-injection, mice were anesthetized with isoflurane and euthanized. Macrophages were isolated by peritoneal lavage in PBS containing 5 mM EDTA, centrifuged at 500× *g* and resuspended in DMEM supplemented with 10% FBS, 2 mM l-glutamine, 50 µg/mL penicillin and 50 U/mL streptomycin. All experiments evaluating apoptosis were carried out in DMEM supplemented with 3% newborn calf lipoprotein deficient serum (NCLPDS), 2 mM l-glutamine, 50 µg/mL penicillin and 50 U/mL streptomycin. Cells were treated (24 h at 37 °C) with combinations of the following: tunicamycin (10 µg/mL from a 1000× stock solution in DMSO), oxLDL (100 µg/mL from a 20× stock in PBS + 0.3 mM EDTA), SEW2871 (1 µm from a 500× stock in DMSO), HDL (50 μg/mL from a 200× stock in PBS with 0.3 mM EDTA), Akt inh. V (10 µm from a 2000× stock in DMSO), LY294002 (10 µm from a 1000× stock in DMSO). Control cells were treated with the corresponding dilutions of solvent/vehicle. For experiments evaluating protein phosphorylation, macrophages were incubated in serum-free DMEM containing 2 mM l-glutamine, 50 µg/mL penicillin and 50 U/mL streptomycin for 1 h prior to addition of stimuli and treatment for the times indicated.

Neutrophils were elicited by intraperitoneal injection of 1 mL of 10% thioglycollate and subsequently followed 12 h later by a second injection of 1 mL of 10% thioglycollate. Three hours later, mice were anesthetized with isoflurane and euthanized and peritoneal cells were isolated by peritoneal lavage with PBS containing 5 mM EDTA, and resuspended in DMEM supplemented with 3% newborn calf lipoprotein deficient serum (NCLPDS), 2 mM l-glutamine, 50 µg/mL penicillin and 50 U/mL streptomycin. 

### 4.7. RT-PCR

Thioglycollate-elicited peritoneal macrophages were isolated as described above. Macrophages were washed with DMEM supplemented with 10% FBS, 2 mM l-glutamine, 50 µg/mL penicillin and 50 U/mL streptomycin. Neutrophils were elicited and isolated as described above. Isolated neutrophils were centrifuged at 500× *g*, resuspended in PBS containing 90% Percoll and purified by ultracentrifugation using a Type 90 Ti Rotor (Beckman Coulter, Mississauga, ON, Canada) as described by others [61]. Total RNA from macrophages and neutrophils were extracted using the RNeasy mini kit (Qiagen, Toronto, ON, Canada) and RNA integrity and concentration were evaluated by measuring the absorbance at 260/280 in a spectrophotometer. In addition, 1 µg of RNA was used to prepare cDNA using the Quantitec reverse transcription kit (Qiagen, Toronto, ON, Canada). Quantitative real-time-(RT-)PCR was performed on prepared cDNA using a mixture of SYBR Green qPCR SuperMix-UDG with ROX (Thermo Fisher Scientific, Ottawa, ON, Canada) and primers listed in Table 1. Quantitative real-time-(RT-)PCR was run on an Applied Biosystems StepOnePlus Real-Time PCR System (Thermo Fisher Scientific, Ottawa, ON, Canada).

### 4.8. Apoptosis

For macrophages treated as described above, cells were fixed with freshly prepared 4% paraformaldehyde in PBS. Fixed cells were stained for apoptotic nuclei by terminal deoxynucleotidyl transferase dUTP nick end labeling (TUNEL) using the ApopTag-Fluorescein In Situ Apoptosis detection kit (EMD Millipore, Etobicoke, ON, Canada). Nuclei were counterstained with 300 nM 4′,6′-diamidino-2-phenylindole (DAPI). Fluorescent images were captured using a Zeiss Axiovert 200M inverted fluorescence microscope with a 40× objective (Carl Zeiss Canada Ltd. Toronto, ON, Canada). Apoptosis was quantified by counting the numbers of TUNEL^+^ nuclei and total numbers of nuclei in 3 fields per well for 4–8 wells per treatment condition and calculating the ratio of the number of TUNEL^+^/total nuclei to determine the ratio of cells that were apoptotic. 

Freshly isolated neutrophils (see above) were plated at 1 × 10^6^ cells per well onto a 96-well plate and either left untreated, or treated with SEW2871 (1 µm from a 500× stock in DMSO) or HDL (50 µg/mL from a 200× stock in PBS with 0.3 mM EDTA) for times indicated. DMSO and PBS/EDTA were added to the same final concentrations to control untreated cells. At the indicated time points, cells were washed with PBS containing 0.1% BSA and 0.1% sodium azide, labeled with rat anti-mouse Ly6G antibody conjugated with APC (BD Biosciences, Mississauga, ON, Canada), Annexin V conjugated with Alexa 488 (Thermo Fisher Scientific, Ottawa, ON, Canada), and 2 μg/mL propidium iodide (Sigma-Aldrich, Oakville, ON, Canada) for 15 min at room temperature and analyzed immediately afterwards on a BD FACSCalibur flow cytometry system. Cell death was quantified by analyzing Annexin V positive staining on Ly6G positive cells.

### 4.9. Electrophoresis and Immunoblotting

Cells were lysed in RIPA buffer (50 mM Tris-HCl pH 7.4 containing 150 mM NaCl, 1% Triton x-100, 1% sodium deoxycholate, 0.1% SDS and 1 mM EDTA) supplemented with protease inhibitors (1 mM PMSF, 1 µP/mL pepstatin A, 1 mg/mL leupeptin, 2 μ/mL aprotinin) and phosphatase inhibitors (phosSTOP, Roche Diagnostics, Mannheim, Germany). Protein concentration was determined using the BCA protein assay kit (Pierce Biotechnology, Rockford, IL, USA). Proteins (30 μg) were separated by SDS-polyacrylamide (12% acrylamide) gel electrophoresis and electrophoretically transferred to polyvinyldifluoride membranes as previously described [62]. Membranes were blocked with 5% skim milk in TBS buffer containing 0.1% Tween-20 for 1 h at room temperature prior to incubation with rabbit anti-mouse phospho-Akt antibody (catalogue number 4060; Cell Signaling Technology, Danvers, MA, USA) overnight at 4 °C. After incubation, membranes were washed and incubated with horseradish peroxidase (HRP)-conjugated goat anti-rabbit secondary antibody (Jackson Immunoresearch Laboratory, West Grove, PA, USA) for 1 h at room temperature. HRP activity was detected using the Amersham Enhanced Chemiluminescence kit (GE Healthcare Life Sciences, Baie d’Urfe, QC, Canada) and band intensities were measured using a Gel Doc imaging system (Bio-Rad Laboratories, Hercules, CA, USA). Membranes were then stripped of bound antibodies using Restore™ Western Blot Stripping Buffer (Thermo Fisher Scientific, Ottawa, ON, Canada), washed and blocked as above, and incubated with rabbit anti-mouse total Akt antibody (catalogue number 9272; Cell Signaling Technology, Danvers, MA, USA) overnight at 4 °C, washed and incubated with secondary antibody as above. HRP-labeled secondary antibody was detected by enhanced chemiluminescence using a Gel Doc Imaging system as indicated above. The band intensities of phospho-Akt were divided by the corresponding band intensities for total-Akt and normalized to a value of 1.0 for unstimulated *WT* or *S1pr1^MWT^* cells.

### 4.10. Statistical Analysis

Results are presented as mean ± standard error of the mean (SEM). Statistical analysis was performed using Prism software (Version 7.02, GraphPad Software Inc., La Jolla, CA, USA). Data from cell culture experiments, involving multiple groups, were subjected to one- or two-way ANOVA followed by the Sidak or Tukey multiple comparisons test as indicated. Data corresponding to analysis of BM-transplanted mice were subjected to the Mann–Whitney Rank Sum non-parametric test. *p* < 0.05 was considered to be statistically significant.

## Figures and Tables

**Table 1:** Quantitative RT-PCR primers for mouse genes.

Target	Forward Primer Sequence	Reverse Primer Sequence
*S1pr1*	5′-ACT TTG CGA GTG AGC TG-3′	5′-AGT GAG CCT TCA GTT ACA GC-3′
*S1pr2*	5′-TTC TGG AGG GTA ACA CAG TGG T-3′	5′-ACA CCC TTT GTA TCA AGT GGC A-3′
*S1pr3*	5′-TGG TGT GCG GCT GTC TAG TCA A-3′	5′-CAC AGC AAG CAG ACC TCC AGA-3′
*S1pr4*	5′-AAC CAA AGA TGT CAG CCA GG-3′	5′-GCA GAA GTCT CCA CGT CCT C-3′
*S1pr5*	5′-GTC GTC CAC TGG AGC ACT G-3′	5′-GTA CAC CAA ATG CCC AGC TT-3′
*Gapdh*	5′-ACC ACA GTC CAT GCC ATC AC-3′	5′-TCC ACC ACC CTG TTG CTG TA-3′

## Abbreviations

Apo	apolipoprotein
BM	bone marrow
DAPI	4′,6′-diamidino-2-phenylindole
ER	endoplasmic reticulum
HDL	high density lipoprotein
HF	high fat
JAK2	Janus kinase 2
KO	knockout
LDL	low density lipoprotein
OxLDL	oxidized LDL
PI3K	phosphatidylinositol-3-kinase
S1P	sphingosine-1-phosphate
S1PR	sphingosine-1-phosphate receptor
SR-B1	scavenger receptor class B type 1
STAT3	signal transducer and activator of transcription 3
TUNEL	terminal deoxynucleotidyl transferase dUTP nick end labeling
